# Supplementary material for: Immunogenicity of an Intranasal Dual (Core and Surface)-Antigen Vaccine Against Hepatitis B Virus Enhanced by Carboxyl-Vinyl Polymer Excipients
Source: Vaccines (Basel). 2025 Apr 25;13(5):464. doi: 10.3390/vaccines13050464 (PMC12115510; doi:10.3390/vaccines13050464)
Supplement: Supplementary file 1 [file vaccines-13-00464-s001.zip › vaccines-3537619-supplementary.pdf]

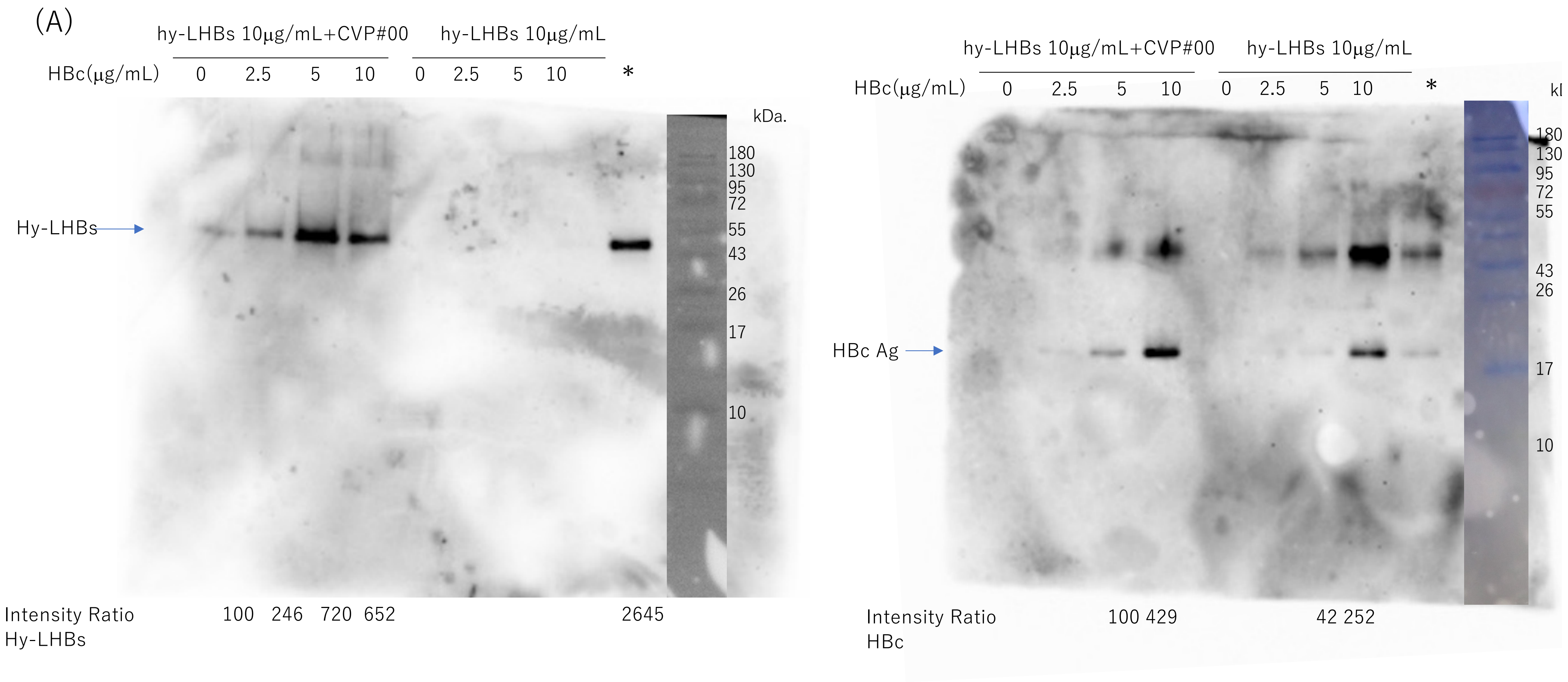

*HBs-Lh 25ng+HBc 25 ng, at -30°C

Figure S1A. Un-clipped gel image of Figure 7A.

(B) 37°C

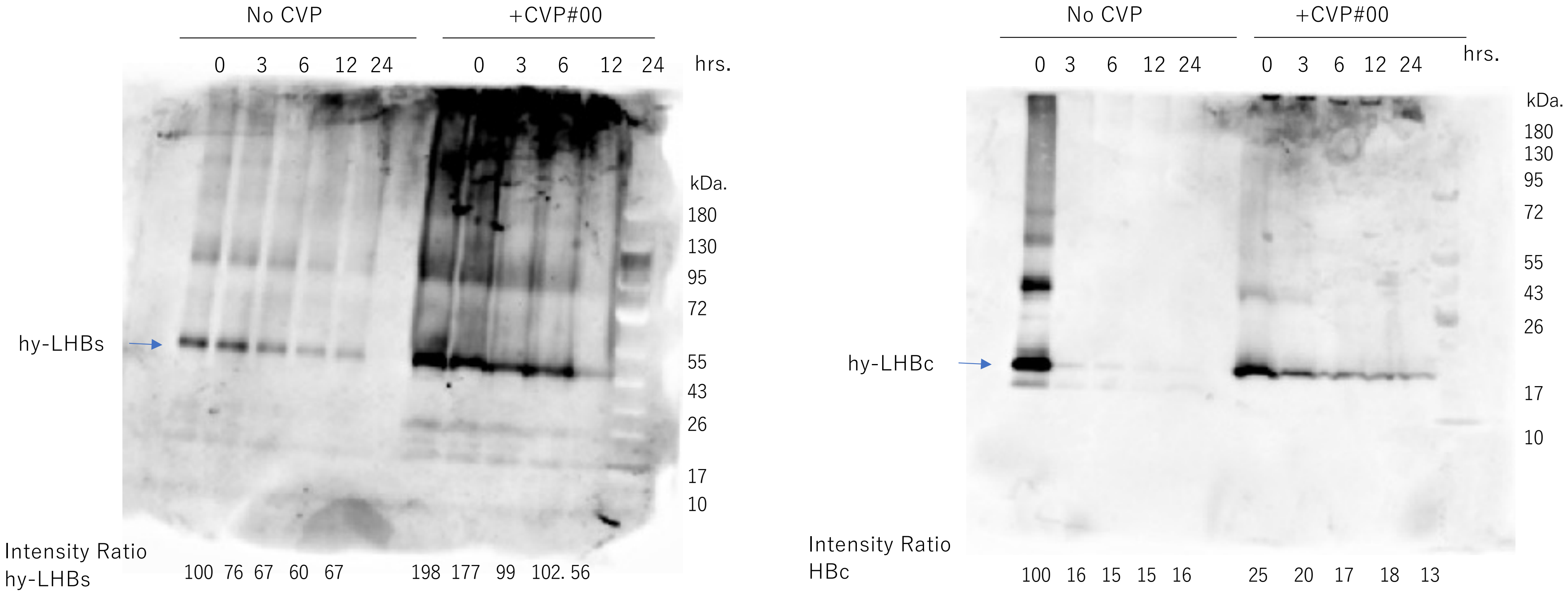

Figure S1B. Un-clipped gel image of Figure 7B.
